# Use and Acceptance of Digital Communication Technology by Older Adults for Social Connectedness During the COVID-19 Pandemic: Mixed Methods Study

**DOI:** 10.2196/41535

**Published:** 2023-08-02

**Authors:** Eric Balki, Carol Holland, Niall Hayes

**Affiliations:** 1 Centre for Ageing and Research Division of Health Lancaster University Lancaster United Kingdom; 2 Nottingham Trent University The Directorate Notthingham United Kingdom

**Keywords:** aging in place, technology acceptance, technology adoption, information and communication technologies, qualitative research, COVID-19 pandemic, Facebook, Meta, WhatsApp, Zoom, generative artificial intelligence, AI

## Abstract

**Background:**

Older adults are at higher risk for health issues, including mental health problems. This was especially apparent during the COVID-19 pandemic, where older adults were simultaneously more vulnerable to the disease and the mental health concerns created by social distancing. Subsequently, the use of digital communication technology (DCT) became a critical option for maintaining social connectedness in older adults. Prior to the pandemic, the low uptake and use of technology by older adults was an established problem, known as the digital divide. However, not much is known about how this may have changed as a result of the pandemic.

**Objective:**

This study aims to explore how older adults maintained social connectedness through DCT during the pandemic and to understand factors influencing the use and acceptance of DCT.

**Methods:**

A mixed methods explorative field study was set up, involving surveys and interviews of 25 community-dwelling older adults (65-88 years old) living in the United Kingdom. The surveys included the internet acceptance questionnaire (based on the Technology Acceptance Model [TAM]); COVID-19 dysfunctional anxiety was captured using the COVID-19 Anxiety Scale (CAS). Background information (demographics, use of technology) was gathered before conducting semistructured interviews. We hypothesized that CAS would affect constructs of TAM and that predictive constructs of TAM would have remained valid during the pandemic. We also posited that there would be unidentified themes outside TAM that impacted the acceptance and use of DCT. We used the quantitative data to guide the semistructured interviews, which were then analyzed through thematic analysis to identify additional themes.

**Results:**

Correlational analysis showed that CAS influences all constructs of TAM. We also saw that the predictive constructs of TAM, especially the perceived ease of use (PEU) and perceived usefulness (PU), remained valid during the pandemic. Common acceptance-influencing themes were encountered in both quantitative and qualitative analyses, with 3 matching the known constructs of TAM (PU, PEU, and behavioral intention). We identified 2 additional themes affecting acceptance, namely influence of the pandemic (situational context) and privacy and security concerns. DCT use (especially email and videoconferencing use) increased during the pandemic, but the results related to social networking sites were mixed.

**Conclusions:**

The COVID-19 pandemic impacted technology acceptance and use by older adults, encouraging their use of certain DCT apps (email and videoconferencing apps, such as WhatsApp). These apps helped insulate them from adverse effects (social isolation and loneliness). Other social networking apps, however, exerted a negative influence, increasing anxiety and a general feeling of negativity. Future studies should maximize older adult agency related to design, privacy, security, and user requirements for development. We also recommend that when studying DCT acceptance for older adults, our additional identified themes should be considered alongside the existing TAM constructs.

## Introduction

### Overview

The health needs of older adults have raised the level of concern, becoming focal points of health policies and initiatives, even prior to the COVID-19 pandemic. The mental health concerns of older adults are particularly insidious and in significant need of improvement. Loneliness and social isolation are especially prolific among older adults [1], both important domains of social connectedness. Loneliness is a subjective perception of a paucity in the quality of one’s relationships and interactions with others, while social isolation represents an objective lack of social connections. A prolonged state of social disconnectedness causes the development of chronic conditions in older adults and mental health problems, including feeling anxious and being depressed [2].

To protect the public, during the pandemic, governments had mandated social distancing. Although these measures protected older adults from being infected, they may have worsened their pre-existing vulnerability to social disconnection [1]. The impact on older adults, who tend to rely on community groups (ie, the church and sports clubs) for socialization, along with close ties to small groups of friends and family, was magnified when compared to their younger counterparts, exacerbating the risk for loneliness [3]. The adverse effects may have been further aggravated by anxiety and negative psychological responses, which have been previously reported during infectious disease outbreaks [4].

Although COVID-19 lockdowns negatively impacted older adults, they provided a unique opportunity to examine how older adults mitigated their potentially worsening isolation. Any emerging solutions from this period could inform and become incorporated into policy guidance and interventions for older adults who may be experiencing isolation and loneliness in normal circumstances.

One such solution was the use of digital communication technology (DCT) for social connectedness. DCT includes digital tools using which 2 or more people communicate with one another through writing, talking, viewing, or listening. A recent comprehensive umbrella review examining the impact of technology on social connectedness showed that DCT offers diverse opportunities for social connectedness in older adults [5]. Such technologies also allow interaction and access to in-person services, such as online shopping and health care. However, technology acceptance, uptake, retention, and use by older adults remain low, with many using them only for short periods [6,7]. In addition, difficulties with actual use (AU) and security concerns [8] may have lowered acceptance [9]. This age-related divide has not improved in the preceding decade, and the pandemic might have further worsened this problem [10].

We examined the circumstantial changes experienced by older adults during the pandemic, their use of DCT, and the evidence for the uptake, acceptance, and adoption of such technologies [11,12]. We explored the perceptions of older adults and their impacts on behavioral intention (BI) and AU. To maintain social engagement through technology during the lockdown, older adults may have made crucial adjustments, providing an opportunity for assessing the potential positive impacts of DCT in the mobility-restricted older population.

Most recent studies examining the impact of technology on older adults’ loneliness and social isolation during the pandemic have been quantitative in nature. Furthermore, in these studies, as the evidence emanated from self-selecting samples [13-16], they generally do not reveal the in-depth reasons for technology uptake or use (or lack thereof) by older adults at a population level. Our paper contributes to the literature by investigating real-world reasons of older adults’ motivations for using technology during the pandemic, especially in the context of remaining connected and the prevention of loneliness, and reports on their perceptions of usefulness, ease of use, and barriers to use.

### Literature Review

Some studies conducted during the pandemic have already demonstrated the benefits of technology for social connectedness in older adults [6,17,18]. Even prior to the pandemic, evidence demonstrated the benefits of technology interventions for social connectedness [5,19]. Indeed, technology use is associated with perceived social capital [20] and may have helped older adults with restricted social mobility to connect more efficiently with their social contacts [21]. DCT also facilitates the development of new relationships, enables continued learning, provides an outlet for personal growth, and is a platform for new hobbies. During the pandemic, DCT might have helped older adults redefine their position in society, mitigating their losses due to circumstances (eg, the inability to see friends and family), creating new roles and retraining to regain lost capacity [22,23], and enabling access to civic services [24], online shopping, and health care [25]. Empowerments such as these are especially important in times of stress and natural disasters [26,27].

Despite the promise shown by DCT, the uptake and acceptance of technology by older adults remain low, resulting in a digital divide [28], when compared to younger adults [29]. A US survey in March 2020 found that only 20% of individuals aged 65 years and older living in the community participated in online social gatherings or virtual parties with friends or family when the government advised significantly reduced social interactions [30]. Thus, many studies adopt age as an explanatory measure of low technology use, ignoring variations in use in later life [31].

Age is associated with many changes in life circumstances that can increase the difficulty in technology uptake and use, such as declining physical and cognitive health, changes in work, and family dynamics. Transitions, such as retirement or bereavement, may also impact technology use in later life [32]. However, because the experiences of older adults are unique and multifactorial, these changes cannot wholly explain the differences in technology acceptance. The picture that emerges from previous attempts to explain low technology acceptance in older adults therefore remains incomplete.

For example, privacy and security concerns are a major consideration in technology acceptance [33,34] but have been underexplored in studies of older adult behaviors [35]. Indeed, the privacy-related decision-making process, also known as the privacy calculus [36], has been extensively studied across mobile apps [37], ecommerce [38], and health-related apps [39] but hardly ever from the older adult perspective. Conventional thought in the literature regards technology acceptance as a relatively predictable process with a similar risk-reward balance leading to similar privacy-related decisions [40,41]. It also suggests that older adults fear privacy-related threats specifically targeted at them, which may deter them from using DCT [42]. Older adults are routinely identified by the media as vulnerable to scams and frauds [43], increasing their anxiety; therefore, there needs to be consideration of how they can exert their agency over their privacy in terms of protection strategies. Older adults often desire to safeguard their personal data and activities [35,44]; however, unreasonable redress of privacy concerns could hinder full participation in digital activities [36,45]. Privacy concerns reported by older users [42] include the misuse of personal information posted online, a lack of trust of online banking [46], and concerns about identity theft, fraud, or bullying online [47]. Social privacy concerns include embarrassment by or conflict with friends and family [48], forwarding of personal information by other users [49], and situational contexts, such as proximity to high-risk situations [50].

Despite the knowledge gaps, the literature reveals 2 emergent explanatory themes for low acceptance of DCT by older adults. One is involuntary limited access caused by a lack of opportunity, linked to socioeconomic status [51]. The other is a voluntary choice to not use technology due to a lack of motivation or interest [52,53] along with emotional experience linked to technology use [54]. However, recent studies have highlighted that the digital divide is now mainly driven by personal preferences and perception of technologies [55] rather than involuntary exclusion, as technology has become increasingly more accessible.

Many studies on the use and uptake of DCT adopt the Technology Acceptance Model (TAM) [11], which describes perceived usefulness (PU) and perceived ease of use (PEU) as the key mechanisms of DCT acceptance/nonacceptance. TAM has gained considerable support in the literature, coinciding with the mass use and adoption of the internet [56-59], and has been applied in widely used tests [60-63]. King and He [62] found that PU is more strongly associated with use intention than PEU but that both factors are important in technology adoption. PU measures how much a user believes DCT will help them achieve a certain goal. A high PU encourages the adoption of DCT by older adults, independently of social pressure [64,65]. TAM predicts that a DCT with high PU and PEU will be favorably adopted. The TAM concept has evolved to include BI as a measure of users’ acceptance of technology [11,66]. PU and PEU can predict a user’s BI to use that technology [52,67], leading to AU. Lee et al [67] modified TAM to include perceived enjoyment (PE) as a motivating factor that further influences BI. Users are also more likely to pursue a course of action that gives the greatest award for the least effort (ie, maximizes the PEU) and will expend the most effort on technology that best improves their circumstances (ie, raises their BI) [64,68]. BI further describes the extent to which a person formulates a conscious decision to use or not use a technology [69], linking it to the person’s behavior and attitude [70].

Some studies have hinted that COVID-19 impacted DCT use, although the precise mechanisms remain unknown [71,72]. It is known that COVID-19 anxiety level increased during the lockdown period [73,74] and was 1 of the dominant mental health symptoms during the pandemic [75]. However, its effect on the use and adoption of DCT remains unaddressed. It may have increased the use and adoption of technology, with older adults seeking out information and answers, but equally could have dissuaded its use due to negative information on the internet that could worsen their anxiety. Therefore, we incorporated COVID-19 anxiety levels in our modeling to answer such questions.

Overall, the literature shows that some aspects of DCT, as well as external factors, dissuade older adults from using technology, although other aspects (both related and nonrelated to DCT) have positive impacts that promote the use of DCT. By studying how older adults used DCT during the pandemic, the variations in technology use, the reasons underlying this use, and whether technology use alleviated their sense of loneliness and social isolation, we can understand why older adults with restricted mobility are sometimes averse to using technology.

### Conceptual Framework

Various studies have highlighted both positive and negative behaviors of technology users seeking to reduce stress or alleviate depressive moods and anxiety. The internet is used for information, video gaming, interacting with friends and family, and online gambling [76,77], helping alleviate the stress of daily living (often referred to as escapism) and avoiding difficult thoughts and problems that could worsen isolation and loneliness. It can be used to access services such as health care and shopping, instilling a sense of “normality” when mobility is restricted.

**Figure 1:**
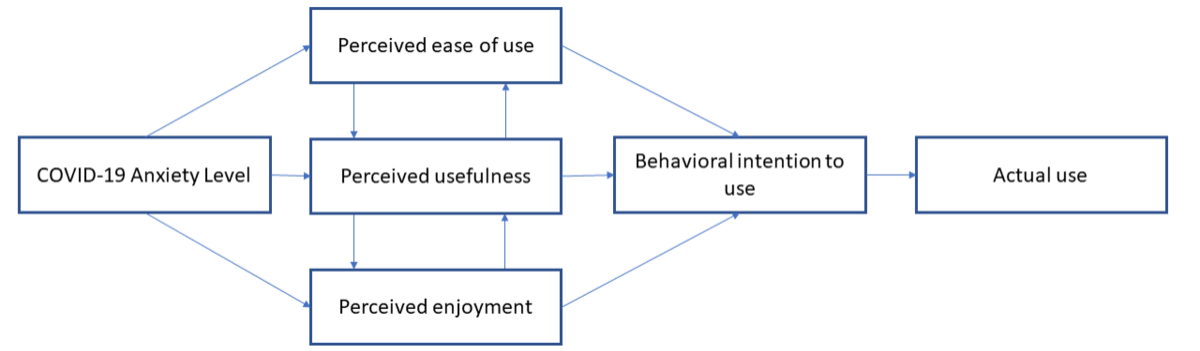
Conceptual model of this research.

This research captured the COVID-19 anxiety levels of older adults and updated TAM. Our aim was to examine the potential influence of COVID-19 anxiety and TAM’s constructs (PU, PEU, PE) on the BI related to DCT use by older adults during the pandemic (Figure 1).

The flowchart in Figure 1 shows the relationships between the research constructs constituting the key determinants of older adults’ intention to use technology. These determinants are the COVID-19 anxiety level, PU, PE, PEU, and BI. By quantitatively and qualitatively investigating the gaps in this model, we could identify other constructs or themes that impact BI and AU.

### Aims

This study was designed to understand the acceptance of DCT during the pandemic and explore the barriers and facilitators to its wider uptake. At the time of the pandemic, we expected adoption to be varied among older adults. It was therefore deemed important to generate insights into the observed differences in DCT uptake by analyzing the relationship between contextual factors related to the pandemic, COVID-19 anxiety and acceptance. We also posited that the COVID-19 pandemic and social distancing would have changed how older adults used DCT. The investigation was conducted to answer the following research questions (RQs):

- RQ1: How did older adults use DCT for social connectedness during the pandemic?
- RQ2: How did COVID-19 anxiety affect acceptance of DCT by older adults?
- RQ3: How did the PEU and PU of DCT influence BI and continued AU of technology during the pandemic, and were there other themes present that are not part of TAM in play?

To bridge the gap in the literature, we aimed to explore the findings and limits of the current knowledge on DCT usage by older adults when physically isolated. We expected COVID-19 anxiety to be negatively correlated to the constructs of TAM (ie, the higher the COVID-19 anxiety, the lower the PU, PE, PEU, and BI related to DCT). We also expected to see that the predictive constructs of TAM would have still been valid during the pandemic (ie, PU, PE, and PEU would impact BI and AU, but there will be other undiscovered themes outside of TAM that need to be discovered). We first set out to test the following hypotheses in the quantitative part of the study:

- COVID-19 anxiety levels are negatively correlated to the constructs of TAM, including PE, PEU, BI, and AU.
- PE and PEU are positively correlated to BI and AU.

To this end, we used the information gleaned from the quantitative results to inform a deeper qualitative exploration and discovery of themes of how DCT use impacted social connectedness during the COVID-19 pandemic.

## Methods

### Ethical Considerations

Ethical procedures aligned with the British Psychological Society consolidated criteria for reporting qualitative research guidelines (COREQ) [78], with ethical approval granted by the Faculty Research Ethics Committee (ref: FHMREC19121).

Prior to obtaining consent, all participants received a study information sheet and were provided with an opportunity to ask questions. Participants were informed of their rights to withdraw from the study at any point and were assured of anonymity.

Prior to starting the interviews, participants were asked to provide written informed consent.

### Study Design

This was a mixed methods explorative field study [12] following a series of quantitative studies that began in April 2020, examining links between social connectedness and technology use during the COVID-19 pandemic [6].

Based on TAM, our study examined various aspects of DCT use. Integrated within these assessments was an evaluation of how the pandemic and DCT use affected social connectedness. We first conducted surveys of participants’ demographical information, DCT use, internet acceptance, and COVID-19 anxiety levels, and then we elicited the perspectives of older adults through semistructured interviews. Applying a phenomenological methodology, we interrogated the data, focusing on individual accounts of experience, coupled with reflexive thematic analysis techniques for analyzing and framing the research data.

### Setting

The study was carried out between November 2020 and April 2021, when restrictions were reintroduced in the United Kingdom and nonessential businesses were closed. People were prohibited from meeting anybody outside their support bubble. The study recruited 25 participants from the northwest of England. The inclusion criteria were (1) community dwelling (ie, aging in place), (2) age 65 years or older, and (3) not cognitively impaired.

### Sampling

Individuals aged 65 years or older were included because the postretirement age is linked to increasing difficulties of aging in place [79], including reduced use of DCT [80]. Participants were recruited through older adult service providers (n=6, 24%), word-of-mouth recommendations (n=12, 48%), and a list of previous participants included in similar studies (n=7, 28%).

One participant was included per household. Of the 34 potential participants, 25 (74%) ultimately agreed to participate in the study. A lack of interest was the main reason provided for nonparticipation.

### Data Collection

Each participant was interviewed over the telephone for 90-150 minutes. The interviews were often split into 2 sessions to prevent disengagement of the participants from the subject matter.

#### Quantitative Data

We gathered information about the DCT apps the participants were commonly communicating with, using the Communications via Internet Checklist survey [81]. To gather information about DCT acceptance, we used a validated, modified version of the TAM questionnaire [11] by Chesney [82], which was created specifically for measuring acceptance among older adults using constructs such as comparison with known products, potential for use, perceived ease of use, PE, and usability [83]. This questionnaire assesses the general acceptance of DCT across 5 primary dimensions: PU, PEU, PE, BI, and AU. The questionnaire includes items related to each dimension, and the participants provided responses (applicability of each statement to their lives) on a 7-point Likert scale (from 1=extremely unlikely to 7=extremely likely). Before completing the questionnaire, the participants were instructed to consider the internet in the broadest sense (inclusive of websites, search engines/tools, email, social media, and videoconferencing).

We also captured the types of DCT used by older adults through the Technology for Social Questionnaire [84]. This allowed us to gather comprehensive information about DCT types that were being used the most and informed open-ended questions later in the qualitative part of the study. We also captured reasons for using/not using DCT to enable us to capture thoughts and perceptions that would advise the semistructured interviews, especially the open-ended questions.

Finally, we captured the anxiety levels associated with the COVID-19 pandemic using the COVID-19 Anxiety Scale (CAS) [85], a 7-item questionnaire on which participants rated the applicability of each statement using a 4-point Likert scale (from 0=not applicable to 3=very applicable). To test our first hypothesis, we carried out a correlational analysis between CAS and the constructs of TAM. To test our second hypothesis, we examined the relationship between the constructs of TAM to validate that previously observed relationships still applied during the pandemic.

#### Qualitative Data

In the first part of the interview, the participants were asked to describe their offline social circle and to mark their emotional closeness to others (eg, family members, relatives, and friends) on a diagram, with concentric circles representing different zones of closeness, based on the method proposed by Antonucci and Akiyama [86], assessing the network size of an individual based on their closeness with network members. They were also asked how they maintained contact with people in the different zones (means of communication deployed) and whether they participated in any organized groups (eg, church attendance). They were then asked a series of questions on how the pandemic had impacted their ability to engage with these groups. Typical questions were “Has your ability to participate in an *organized group* been affected by the COVID-19 pandemic?,” “How have you communicated with other members of this group during the COVID-19 pandemic?,” and “How has DCT helped or hindered you in communicating with other members of this group during the COVID-19 pandemic?” Interviews were partially retrospective, seeking to explain how the pandemic impacted their daily living and whether the technology used for social connectedness met their expectations.

In the second part of the interview, we explored the participants’ perceptions and use of DCT for social connectedness. Here, respondents provided their opinions on different apps and described their use. Typical questions were “Is there anything that prevents you from using a *DCT type* more actively than you currently do?” and “What is your general perception of the *videoconferencing tool type*?,” probing whether the respondents liked or disliked a particular app and asking them to elaborate. To probe how the pandemic impacted use, we asked questions such as “How has your use of *email* changed during the COVID-19 pandemic?” and “Do you think the *videoconferencing application type* has helped you feel less lonely during the COVID-19 pandemic?” The participants were provided with an opportunity to elaborate. At this time, the participants were usually given a break before resuming the interview.

The third section of the interview consisted of open-ended questions designed to prompt lateral thinking based on information gathered from the quantitative part of the study. Typical questions were “What is technology for you? What does the word ‘technology’ bring to mind?” and “Do you think that you have started using communication technology more during the pandemic?” The participants were prompted to mention the benefits, concerns, social influence, perceived need, barriers, facilitators, stigmatization, and costs of technology. Other questions in this section were based on the results of the internet acceptance questionnaire, namely whether PU, PEU, and design had influenced participants’ use of the technology. Questions such as “Does the design of the *DCT* *type* you use impact your ability to use it?” enabled probing the usability, ease of use, design, and enjoyment aspects of technology. Other questions focused on privacy and security, such as “Do your privacy and security concerns prevent you from using any *DCT type*?” and probing whether such concerns became heightened during the pandemic.

The last part of the interview focused on specific anxieties related to the COVID-19 pandemic. This part was intended to holistically capture the participants’ mindset regarding the pandemic. Drawing on the CAS results and other quantitative data, we posed questions such as “Have you felt that since the COVID-19 pandemic your use of technology has changed?” and “Has COVID-19 impacted your use of a *DCT type* for connecting with friends and family?”

All interviews were recorded and transcribed verbatim. A summary of the interview was sent to each participant to check for any misinterpretations. No participants responded to the summary.

### Data Analysis

The quantitative data were entered into IBM SPSS Statistics 28.0 software to create descriptive statistics of the participants. The reliability of the questionnaire for each construct was measured using Cronbach α. The reliabilities of all 5 DCT measures exceeded the recommended minimum standard of 0.60 (PEU=.71, PE=.71, PU=.74, BI=.72, AU=.71). The sixth variable was COVID-19 anxiety measured on CAS (Cronbach α=.88) [85]. Pearson correlational analysis examined whether there was a link between CAS scores and PU, PEU, PE, and BI. The outcomes of the analysis from the quantitative data were then collated to identify themes for designing the open-ended probing questions of the semistructured interview.

All transcripts from the interviews were manually checked for anonymity before importing into QSR NVivo version 12 for analysis. To ensure a consistent coding approach, we coded 5 transcripts at the start of the data analysis and discussed issues of salience raised by the participants among the research team. As the interviews were coded by a single researcher, we did not calculate the intercoder reliability or quantify the agreement [87] but rather focused on the impression of important topics when coding a text passage.

Using NVivo, inductive codes were attached to quotations relevant to the RQ. During this process, factors described in Balki et al’s [5] umbrella review were used as sensitizing concepts, such as using terms to denote various typologies of DCT. We used an inductive-deductive reflexive thematic analysis approach informed by Braun and Clarke [87], with hypotheses defined at the start of the quantitative part of the study. Coding was detailed; in many cases, multiple codes representing different factors influencing technology use were attached to quotations. The coded transcripts were discussed within the research team. Through this collaboration, new codes were added, overarching categories of codes were formed and refined, and a model of the findings was shaped. The entire process took 12 weeks. Within the last 2 weeks, few new codes were added, indicating that data saturation had been reached. The themes and subthemes were therefore developed based on the participants’ narratives.

## Results

### Quantitative Results

#### Sample Descriptives

The sample consisted of 25 participants aged 65-92 years. The average age was 73 (SD 3) years, and 72% (n=18) of the participants were women. Just over 32% (n=8) of the participants lived alone.

#### Types of DCT and Frequency of Use

Table 1 lists the most common types of DCT used by the participants. Almost all participants had access to mobile or smart phones, and the majority had access to some kind of tablet.

We also identified the types of DCT that participants dedicated to social connection (Table 2). WhatsApp and Zoom were most frequently used for this purpose, followed by various online forums.

**Table 1:** Types of DCT^a^ frequently used by the participants (N=25).

Type of DCT	Participants, n (%)
Mobile phone	23 (92)
Text messaging/WhatsApp/Skype	22 (88)
Email	19 (76)
Voicemail	18 (72)
Videoconferencing	13 (52)
SNS^b^	12 (48)
iPad/tablet	11 (44)

^a^DCT: digital communication technology.

^b^SNS: social networking sites.

**Table 2:** DCT^a^ types used for social connectedness and numbers of frequent users (N=25).

Type of DCT	Participants, n (%)
Zoom/WhatsApp	20 (80)
Online discussion	17 (68)
Twitter	14 (56)
Facebook/Meta	9 (36)
LinkedIn	9 (36)
Instagram	6 (24)
Online dating	4 (16)
Pinterest	3 (12)

^a^DCT: digital communication technology.

#### Reasons for Using DCT

We gathered data on the purposes for which older adults adopted DCT and the types of apps availed for those purposes (Table 3). Notably, older adults obtained much of their information and news during the pandemic from social networking sites (SNS) and used videoconferencing apps mainly to connect and communicate with family members and friends.

**Table 3:** Reasons for using DCT^a^ by the participants (N=25).

Type of use	Email, n (%)	Videoconferencing, n (%)	SNS^b^, n (%)	None of these, n (%)
Connect/communicate with family members	12 (48)	21 (84)	23 (92)	2 (8)
Connect/communicate with friends	12 (48)	18 (72)	8 (32)	6 (24)
Document/update others on one’s daily life	3 (12)	11 (44)	16 (64)	9 (36)
Express opinions on political issues	6 (24)	2 (8)	17 (68)	8 (32)
Find a new hobby/support an existing hobby	7 (28)	3 (12)	11 (44)	14 (56)
Find a romantic partner	3 (12)	2 (8)	4 (16)	19 (76)
Get health-related information or advice	9 (36)	9 (36)	8 (32)	14 (56)

^a^DCT: digital communication technology.

^b^SNS: social networking sites.

#### Reasons for Not Using DCT

We captured data on participants’ reasons for not using DCT during the pandemic (Table 4). Most commonly, the participants reported either that their family members were not using DCT or that the method (eg, email) was too impersonal. Unreliable information and privacy concerns about SNS also emerged as dominant reasons for not using DCT.

**Table 4:** Reasons for not using DCT^a^ by the participants (N=25).

Reasons for not using DCT	Email, n (%)	Videoconferencing, n (%)	SNS^b^, n (%)	None of these, n (%)
I have a disability, which makes it difficult for me.	0	0	0	25 (100)
I have friends and family who go online for me.	0	0	0	25 (100)
I never learned how to use it.	0	0	0	25 (100)
It is too expensive (including getting broadband).	0	4 (16)	3 (12)	21 (84)
It is too complicated or difficult to use.	11 (44)	7 (28)	9 (36)	13 (52)
It is too slow.	3 (12)	14 (56)	7 (28)	12 (48)
I am concerned about security.	13 (52)	4 (16)	18 (72)	5 (20)
Most of my friends do not use it.	9 (36)	6 (24)	15 (60)	5 (20)
I am concerned about privacy.	11 (44)	7 (28)	17 (68)	4 (16)
The information shared is not reliable	16 (64)	2 (8)	19 (76)	4 (16)
I do not like this method because it is impersonal.	14 (56)	5 (20)	16 (64)	3 (12)
Most of my family does not use it.	11 (44)	7 (28)	16 (64)	3 (12)

^a^DCT: digital communication technology.

^b^SNS: social networking sites.

#### CAS Scores

The overall mean CAS score of the sample was 14.96 (SD 5.36), with 15 (60%) surveys scoring above 16, showing the anxiety levels were high in our sample. We divided the sample into 2 groups: those living alone (n=6, 24%) and those living with a partner (n=19, 76%). This allowed us to be confident that living alone did not impact the levels of anxiety resulting in any skew. An independent-sample *t* test comparing the mean CAS scores of the participants living alone (16.34, SD 3.20) and those not living alone (11.34, SD 3.82) identified no significant difference (*P*>.05) between the groups.

#### Perceived Usefulness, Perceived Ease of Use, Perceived Enjoyment, Behavioral Intention, and Actual Use of DCT

We used descriptive statistics to capture the participants’ perceptions of PU, PEU, PE, BI, and AU related to DCT. To identify whether they generally felt positive or negative about a TAM construct related to DCT, we divided scores into 2 ranges, high and low scores. Table 5 summarizes the frequencies of the participants’ scores based on their responses to the 5 constructs of the TAM questionnaire. PU scores ranged from 8 to 22 (mean 14.40, SD 5.79). Participants scoring between 8 and 16 were classified as feeling negative about PU, whereas those scoring above 16 were classified as feeling positive. Of the 25 participants, 52% (n=13) provided scores in the 8-16 range, indicating a slight negative bias against PU. In contrast, 56% (n=14) of the PEU scores ranged from 16 to 25, showing a positive bias toward PEU. PE was also positively rated, with 56% (n=14) of the scores in the 16-24 range. Although only 44% (n=11) of the participants showed a positive BI, 56% (n=14) provided positive AU scores, indicating a slight discrepancy between BI and AU. The data informed us of the role the 5 constructs play in DCT use and allowed us to use them for deeper exploration behind the causes of both negative and positive perceptions of TAM constructs further in the qualitative part of the study.

Table 6 lists the Pearson correlations between pairs of participants’ scores on CAS, PU, PEU, PE, and BI to explore the impact of COVID-19 anxiety on the constructs of TAM for our first and ssecond hypotheses.

**Table 5:** Descriptive statistics.

TAM^a^ construct and perception			Minimum		Maximum	Mean (SD)		Scores		Frequency, n (%)
**PU^b^**										
	Negative	8		22		14.40 (5.79)	8-16		13 (52)	
	Positive	N/A^c^		N/A		N/A	16-22		12 (48)	
**PEU^d^**										
	Negative	7		25		14.92 (5.89)	7-16		11 (44)	
	Positive	N/A		N/A		N/A	16-25		14 (56)	
**PE^e^**										
	Negative	8		24		14.84 (6.12)	8-16		11 (44)	
	Positive	N/A		N/A		N/A	16-24		14 (56)	
**BI^f^**										
	Negative	4		8		5.44 (0.97)	4-5		14 (56)	
	Positive	N/A		N/A		N/A	5-8		11 (44)	
**AU^g^**										
	Negative	4		8		5.64 (1.18)	4-5		11 (44)	
	Positive	N/A		N/A		N/A	5-8		14 (56)	

^a^TAM: Technology Acceptance Model.

^b^PU: perceived usefulness.

^c^N/A: not applicable.

^d^PEU: perceived ease of use.

^e^PE: perceived enjoyment.

^f^BI: behavioral intention.

^g^AU: actual use.

**Table 6:** Correlational analysis between variables (N=25).

TAM^a^ construct	Pearson R					
	PU^b^	PEU^c^	PE^d^	BI^e^	AU^f^	CAS^g^
PU	1	0.928^h^	0.932^h^	0.848^h^	0.913^h^	−0.891^h^
PEU	0.928b	1	0.960^h^	0.845^h^	0.854^h^	−0.837^h^
PE	−0.932^h^	0.960^h^	1	0.855^h^	0.910^h^	−0.867^h^
BI	0.848^h^	0.845^h^	0.855^h^	1	0.839^h^	−0.749^h^
AU	0.913^h^	0.854^h^	0.773^h^	0.839^h^	1	−0.855^h^
CAS	−0.891^h^	−0.837^h^	−0.867^h^	–0.749^h^	−0.855^h^	1

^a^TAM: Technology Acceptance Model.

^b^PU: perceived usefulness.

^c^PEU: perceived ease of use.

^d^PE: perceived enjoyment.

^e^BI: behavioral intention.

^f^AU: actual use.

^g^CAS: COVID-19 Anxiety Scale.

^h^*P*<.01 (2-tailed).

##### Hypothesis 1: COVID-19 Anxiety Will Negatively Impact TAM Constructs

Table 6 reveals significantly negative relationships between CAS and internet (DCT) acceptance constructs: CAS versus PU (R=−0.891, *P*<.01), CAS versus PEU (R=−0.837, *P*<.01), CAS versus PE (R=−0.867, *P*<.01), CAS versus BI (R=−0.749, *P*<.01), and CAS versus AU (R=−0.855, *P*<.01). Therefore, we can assume that the more COVID-19 anxiety a person experiences, the lower their PU, PEU, PE, BI, and AU, confirming our first hypothesis.

##### Hypothesis 2: Predictive TAM Constructs Remained Valid During the Pandemic

Conversely, BI and AU were positively correlated with all internet (DCT) acceptance variables, confirming that PU, PEU, and PE all contribute to BI and AU in older adults, confirming our second hypothesis.

### Qualitative Results

#### Emergent Themes

During the interviews, all participants expressed that they were experiencing major changes due to the COVID-19 pandemic and were no longer able to engage in the in-person activities they used to engage in before the pandemic. Predictably, most participants (n=23, 92%) expressed an increase in loneliness and social isolation.

It’s really difficult to make contact with friends I used to meet on a regular basis before the pandemic. Now my day is spent at home on my chair.

Participant 8 (P08)

Since the pandemic, you tend to feel a lot lonelier, isolated from the rest of the world. I can’t go out, meet family or friends…it can get very depressing.

P11

The coding analysis of the interviews revealed 5 major themes that impacted technology use for social connectedness, 3 of which match known themes of TAM, namely PU, PEU, and BI; 2 additional themes were identified, demonstrating that DCT acceptance by older adults (especially during the pandemic) cannot be determined from TAM analysis alone. These included privacy and security concerns, and situational context (impact of the pandemic).

##### Perceived Usefulness of DCT

The PU of DCT partly depended on how and whether the participants had used DCT before the pandemic. Participants expressed 3 main drivers of DCT use: perceived need, general interest, and willingness to invest time and effort. The most discussed DCT types were email, videoconferencing, and SNS (eg, Facebook/Meta). Videoconferencing apps accounted for most of the positive PU ratings, with the majority (n=22, 88%) of the participants reporting benefits from these apps (eg, WhatsApp, Zoom). Although the use of these tools did not measure up to in-person activities for older adults, they allowed engagement with friends and family during the pandemic, which was valuable to the participants.

I love the quick and easy on-demand nature. We just call people, and sometimes they pick up and sometimes they don’t if they are busy, but the ability to do that is amazing.

P14

These days, yes definitely, it’s the closest thing you have to meeting someone in-person, so definitely helps.

P23

Some participants commented that videoconferencing enabled access to services such as health care, which were usually visited in person:

Well, it saves us the time going for the (GP) visit. But you know it's an hour’s journey both way[s], so used to take a lot of time.

P09

When queried on the usefulness of email during the pandemic, the participants provided mixed responses. Many expressed concerns with receiving spam and bills through email:

I tend to get a lot of spam. That’s something I do dislike. The other thing I dislike is important messages ending up in the junk folder.

P03

Well, these days you don’t like receiving bills. Our bills have increased a lot during the pandemic as we are spending a lot of time at home.

P12

However, many participants expressed a positive overall perception of email:

Imagine the days when we would be sitting in front of a typewriter typing up letters. Those days are gone, and it’s made things so much more efficient.

P19

Most participants (n=22, 88%) received and sent emails in equal proportion, demonstrating that they were active users of email, with interview responses confirming this. In contrast, some participants (n=16, 64%) were sporadic users of Facebook/Meta, with only 36% (n=9, ) of the interviewed participants mentioning that they were active users, contrary to our expectations. We also noted that participants generally found Facebook/Meta less useful than their earlier perceptions of it. As many as half of participants commented that their Facebook use had diminished over time:

I think definitely as time is passing, my use of Facebook(Meta) is diminishing. I saw the value of it years ago, but now I don’t.

P11

I think I have shied away from Facebook. I was starting to dread switching it on. I think now I feel like that’s how it was feeling.

P17

Two main reasons for the perceived lack of usefulness emerged: a perceived increase in pandemic-linked information volume on their newsfeeds and pages that increased their anxiety. After associating Facebook/Meta with negative feelings, the participants began avoiding the platform. Moreover, their previous reasons for using the app, such as knowledge -seeking or connecting with family members, had lost their potency:

You get is (sic) lots of news stories that you don’t want to read. Hence, it’s not very friendly, nor enjoyable.

P13

Others hinted that Facebook/Meta had become less useful, not because of the pandemic but because of diminishing use by younger family members:

My grandchildren used to put up pictures before, so it used to be interesting, but they don’t really use it anymore.

P16

Overall, we found that the perceived usefulness of a DCT depended on how it was being used, with some embracing it more than others.

##### Perceived Ease of Use of DCT

The ease of use and design was an important influencer. Participants mentioned that the difficulty level of a DCT determined whether they used or abandoned the technology. Comments related to WhatsApp were largely positive:

It’s (WhatsApp) easy to use and quite intuitive.

P24

Many participants expressed ease-of-use issues with Facebook/Meta, which has undergone many design changes over the years. For instance, the addition of newsfeeds and shopping increased the difficulty of navigating the platform by older adults:

It’s (Facebook/Meta) just so complex…it used to be about sharing pictures of family and friends, and now, it has shopping and news and other things. Too much for me to manage.

P18

In addition, 72% (n=18) of the participants expressed that the PEU of a technology influenced their intention to use the technology (ie, their BI) during the pandemic. Negative perceptions of certain apps (eg, Facebook/Meta) may have prevented frequent use. On the contrary, easy-to-use apps (eg, WhatsApp) were more commonly used during the pandemic than before.

##### Behavioral Intention of DCT Use

To establish the changes in participants’ intentions to use DCT, we first ascertained the kinds of organized groups and events that the participants engaged in before the pandemic, providing a prepandemic perspective of social life. Many participants expressed that previous social groups were no longer operating.

My wife and I used to go to the local bingo or play cards there. But that has not been possible.

P13

Before the pandemic, it would be getting together for dinners and drinks, card and poker games.

P07

Participants mentioned that some of their group interactions continued online, but when questioned whether the online activities increased their use of DCT, their responses were mixed. One participant replied:

I used to attend the church before. I am not a very religious person, but I would go, say, once a month. But since the pandemic, it’s now gone. I know they moved some of the congregation online, but it doesn’t feel the same.

P04

However, many participants began using DCT to maintain the types of activities they previously engaged in:

Before the pandemic, I was part of a card games group. We would go over in each other’s houses and play that; we have since then started playing online.

P17

We found that 68% (n=17) of the participants continued to interact with their offline social groups through DCT, influencing their BI. We further explored the perceived impact on loneliness and social isolation, that is, whether their BI was influenced by the need to engage with others. Overall, 76% (n=19) of the participants reported that email helped them maintain their social connections and reduced loneliness:

I think it has in fact a bit of a calming influence during the pandemic. My face lights up when I see an email from a family member.

P16

However, when asked similar questions about Facebook/Meta, participants provided dramatically different responses. Many participants commented that Facebook/Meta worsened or did not affect their sense of loneliness. Only 36% (n=9) of the participants commented that Facebook/Meta helped them remain connected. We found that Facebook/Meta avoidance by participants was exacerbated not only by the pandemic but also by the evolution of the social media site over time:

If anything, it (Meta) makes you lonelier. When you keep getting newsfeeds of negativity.

P03

Among the participants who reported that Facebook/Meta improved their connectedness and reduced their loneliness, interactions with friends and family was a major theme:

When the grandkids do put up pictures, it’s nice. It’s also nice when we comment, and they respond back. So, in that sense, maybe the answer to your question is yes, it does from time to time make us feel less lonely.

P05

Participants who reported that videoconferencing apps reduced their loneliness also reported increased use of those apps:

I am glad I have started using it (videoconferencing) more as it saves thinking about catching the train or driving to meet someone.

P04

However, there were some dissenting voices. Some participants were reminded of the pandemic when speaking to loved ones via video rather than meeting them in person:

Bizarrely enough, sometimes doing videoconferencing, the aftermath is that you feel lonelier not being able to see loved ones in person. So, yes, I would say I have become lonelier during the pandemic.

P06

In general, the participants realized that videoconferencing could not fully replace face-to-face interactions:

It’s (videoconferencing) allowed us to maintain connections. I think deeper connections only come from spending time together in person. I think technology allows us to remain connected, and it’s good to talk but doesn’t replace the real thing.

P15

#### Impact of the Pandemic on DCT Use

The pandemic increased the frequency of DCT use by the participants: 76% (n=19) stated that their use increased. Participants frequently mentioned challenges that were directly related to the impact of the pandemic on their lives, influencing their DCT use. In particular, the psychosocial turmoil caused by the pandemic changed the participants’ behavior:

I think it’s been particularly hard during the pandemic. Before we would get the grandkids over, and they would come and stay with (sic), for example, during Easter. So, it’s felt lonelier now than it did before.

P03

Some participants mentioned that isolating with just 1 other person (usually a family member) had possibly exacerbated the situation by increasing their sense of loneliness:

I think just having my son’s company has made me feel lonelier. Before the pandemic, I at least was able to get together with my close friends.

P01

This viewpoint was particularly obvious when participants were in a caregiving relationship. Such isolated people sought online forums and groups that allowed them to connect with others, which they would not have joined under normal circumstances:

Not being able to things I would have normally [done], I now speak with my friends that I used to play card games with online through Zoom, and we even are able to play online.

P07

Overall, the pandemic impeded the ability of older adults to maintain connections outside their immediate social circle and appeared to degrade the perceived quality of existing connections. Although DCT helped maintain these connections, it was not deemed an adequate replacement. However, 24% (n=6) of the participants viewed the pandemic as an opportunity to make new contacts or to reconnect with lost previous contacts:

Have been getting in touch with old friends from university during the pandemic, which has been nice.

P09

Two types of DCT especially benefited from the pandemic: videoconferencing and online shopping:

Our use of WhatsApp and video calls has gone up so much since the lockdowns; we used to hardly use it before.

P16

Pretty much all of our shopping is now being done online; they drop it outside for us on our doorsteps. It makes it easier to order, but then we are not able to choose the quality of vegetables.

P22

Of the participants, 50% (n=13) expressed DCT privacy concerns directly related to the pandemic. We explored the general fears regarding the privacy and security of technology as a separate theme.

### Impact of Privacy and Security Concerns

Privacy and security concerns around DCT usage emerged as a major influencer of participants’ technology-related attitudes and beliefs. The participants expressed strong opinions on this theme.

The use of Facebook/Meta raised the highest security and privacy concerns. Several mainstream media stories reported privacy breaches and ethical issues revolving around Facebook/Meta at the time of data collection, which may have influenced the participants’ responses:

Privacy is a conversation technology companies (like Meta) avoid. We become dependent on their technologies, and here they are not even telling us properly what data they are gathering on us and what are they doing with it.

P01

Others were concerned with targeted advertising on Facebook and its potential link to privacy breaches:

Bizarre things like unrelated adverts appearing. When I mentioned needing toilet paper to my sister (on WhatsApp) and then logged into Facebook, adverts for where I can get toilet paper started popping up!

P19

Of the 25 participants. 72% (n=18) expressed privacy and security concerns around technology usage. Fears included unauthorized access to or use of their information, illegitimate activities, and compromise of personal information, such as information falling into the wrong hands. In general, the participants were concerned about their data with Facebook/Meta:

What if it all got hacked and all the information they on you went into the wrong hands. Like when we get spam messages, what if they use the data on us [to] really create a convincing spam fraud?

P12

## Discussion

### Principal Findings

Overall, our findings revealed a complex picture of older adults’ interaction with DCT. We confirmed that pandemic-related social distancing measures increased COVID-19 anxiety and that it was correlated to known constructs of TAM. We also found that the predictive constructs of TAM remained valid during the pandemic. Some DCT apps worsened well-being, potentially raising the participants’ COVID-19 anxiety and exacerbating negative perceptions of technology. Other DCT types insulated older adults from the impact of isolation, especially by helping them find information, access services, and connect socially. Five themes—PU, PEU, BI, impact of the pandemic (situational context), and privacy concerns—emerged; these require special attention for those seeking mitigating actions against the detrimental consequences of home confinement. Our findings can also be used to guide DCT design and help alleviate known problems linked to the usability of DCT apps.

Our first RQ dealt with how participants used DCT for social connectedness during the pandemic. Most participants were primarily trying to replicate online their in-person activities before the pandemic, including connection with friends and family, normal social interactions, accessing primary care services (eg, general practitioners), and shopping.

The COVID-19 pandemic has changed the digital profiles of older adults, accelerating the acceptance, adoption, and adaptation of certain digital technologies. DCT became an integral part of older people’s lives and was used more frequently during the pandemic. This increased usage was followed by an improved perception of the benefits of DCT.

In the quantitative part of the study, the participants were required to select their reasons for using or not using DCT (SNS, email, videoconferencing) from a list (Tables 3 and 4). The main reasons for avoiding SNS were privacy concerns, the impersonal nature of SNS, and nonuse by friends and family. SNS were used for connecting with family and friends, expressing opinions on political issues, and staying updated with news and current affairs. Videoconferencing was mainly used for communicating with friends and family. Our thematic-based breakdown allowed deeper insights into older adults’ motivations for DCT use. The participants were guided by their perceptions of whether they needed DCT, whether they were interested in the types of DCT, and their willingness to invest time and energy.

We found that older adults desired to be more communicative, informed, and have better access to services that could not be visited in person. These desires influenced their BI to use DCT. DCT buffered older adults from exclusion, social isolation, and loneliness; kept them informed about current activities; and provided access to civic services. Older adults’ desire to communicate increases in times of stress and natural disasters in order to tackle social isolation and loneliness [26,27], seek out information [88,89], and access civic and health care services [24].

Our second RQ probed the effect of COVID-19 and its associated anxiety on the constructs of TAM. The hypothesis related to quantitative data revealed that COVID-19 anxiety directly and significantly affects all predictive constructs of TAM. This was then qualitatively explored through our semistructured interviews with open-ended questions. Certain DCT apps were more popular than others (eg, WhatsApp was adopted more enthusiastically than Meta/Facebook), confirming that older adults relied on DCT to remain socially connected and occasionally to build and maintain new contacts while staying safe. This continued connectivity may have alleviated some of their COVID-19 anxiety. Equally, our participants revealed that certain information they found on social media sites, such as Facebook/Meta, may have increased their anxiousness about the pandemic. Discourse around privacy and security (and lack thereof) on social media may have further impacted COVID-19 anxiety.

COVID-19 itself impacted both how DCT was being used and its frequency, with higher levels of technology use for everyday activities, such as looking for information, shopping, socializing, and entertainment. Older adults adopted well to the pandemic, with many increasing their technology use, and while the longer-term impacts of the pandemic are yet to be determined, it clearly had a major impact on helping bridge the digital divide between younger and older adults. This was contrary to common stereotypes of older adults being unable to adapt to new types of technology use and appears to indicate that the motivation to remain connected during the pandemic accelerated the acceptance of DCT. These findings suggest that the unique combination of factors during the pandemic, namely a constraint on previous means of social behavior and the ability to access and use commonly available technology, was important to technological adoption by older adults, especially in the context of the pandemic.

Third, we investigated how the constructs of TAM and other emerging themes influenced the BI and AU related to DCT. Our quantitative results revealed that PE and PEU impacted older adults’ BI and AU related to DCT during the pandemic, supporting the results of earlier studies [61,62] and demonstrating that the validity of TAM remained during the pandemic. Independently, in the qualitative exploration, we extracted 3 of the 4 commonly known TAM constructs that impact AU (PEU, PU, and BI) as themes. Viewing the qualitative data through the TAM lens, we associated certain technologies (videoconferencing apps and email) with high PEU and PU and certain other technologies (social media apps, such as Facebook/Meta) with negative perceptions. Both email and videoconferencing apps became important tools for social connectedness, especially with loneliness reduction, being favorably assessed by an overwhelming majority of the participants (over 76%). Videoconferencing was favored over email for social connectedness, especially as a replacement for in-person contact during social distancing.

However, in some instances, videoconferencing apps reminded the participants of the absence of in-person connections (often with geographically close loved ones). Although such personal disconnect may have worsened their loneliness, the overall consensus of PU was overwhelmingly positive, supported by the participants’ responses during interviews. Apps such as WhatsApp and Zoom were perceived positively, with special appreciation of the PEU and design of WhatsApp. The limitations of some videoconferencing apps (eg, the number of video users permitted in WhatsApp) encouraged some participants to seek alternative applications (eg, Zoom) and others to abandon DCT. These results highlight the importance of the negative impact of design-related flaws on BI and AU.

Our study confirmed the importance of PEU and design in the acceptance of DCT. The PEU and design of Facebook/Meta were viewed unfavorably: the perception of the participants was that as the platform has evolved, it has become more cluttered and confusing than when they first started using it. We conjecture that design was a major reason the participants found it difficult to engage with Facebook/Meta. The pandemic also coincided with the accelerating volume and frequency of information on Facebook/Meta, much of it related to the pandemic. The PU of Facebook/Meta severely diminished, likely because many participants found this platform a negative space to occupy. In particular, Facebook/Meta increased the participants’ anxiety and was ineffective for social connectedness for many participants.

This finding elucidates a potential mechanism by which the contents of some DCTs (social media and SNS) influence the PU of those DCTs. Some participants reduced their use of Facebook/Meta when their grandchildren (major motivator of DCT adoption) moved from Facebook/Meta to other social media sites, such as Instagram (incidentally also owned by Facebook/Meta), which are inaccessible or unfamiliar to older adults. Although our study provided strong evidence for diminished BI and AU related to Facebook/Meta in older adults, it did confirm Lee et al’s [67] conjecture of PU and PEU being important constructs of TAM as motivating factors that influence BI. The main theoretical basis of the TAM approach suggests that an individual’s acceptance of a technology is influenced by their intention to use it, and this, in turn, is influenced by a set of core beliefs about the benefits and outcomes of its use. A weakness of TAM is the absence of affective or motivational factors whose effects are considered behaviorally relevant [90]. Our study found that BI was most affected by content and design, which in turn could have impacted motivational factors as well.

An age-friendly design also encourages the use of technology. The lack of age-friendly designs may be preventing older adults from fully using the functionality of certain DCTs, such as Facebook/Meta. All design processes should aim to deliver value or worth to their users [91,92] and create a service that users will perceive as useful. Indeed, involving users in the design process to understand their value is a well-established concept in product design but is seldom applied to older adults and design of technology. Gould and Lewis [92] mentioned the need for understanding users when designing a new product but noted that such recommendations are commonly ignored by designers who consider their own experience more relevant. Older adults should be involved in the design process because they are less likely than younger users to adopt the most current version of a DCT or succumb to social pressures [64,65]. Unlike general and easy-to-use email and videoconferencing apps, which are designed for users of all ages, social media platforms, such as Facebook/Meta, have always targeted younger users and are constantly evolving to attract and retain those users at the risk of losing older ones.

Older adults use DCT when it fulfills a perceived need in their lives. Under the pandemic restrictions, these needs were modified and the DCT use changed accordingly. DCT enabled older adults to maintain their social connectedness and interactions with service providers, such as health care. Participants also noted an uptick in the volume of emails, which improved the sense of connectedness for some participants. Furthermore, older adults with restricted mobility frequently used DCT to facilitate shopping delivery to their homes, demonstrating the evolution of online shopping. Usage of apps such as WhatsApp and using video provided some resemblance of in-person contact.

### Comparison With Prior Work

Previous studies on PU have found that a significant emotional connection between technology and older adults [54] is pivotal for the adoption of new and existing technologies [53]. Such connections can improve the quality of life of older adults [16]. We lacked prepandemic data on PU but were able to infer an increase from our qualitative data, with the participants expressing opinions that they found certain apps more useful during the COVID-19 pandemic than they had before.

Studies on DCT motivation have pointed out that under certain circumstances, older adults may become sufficiently interested to learn a particular aspect of DCT [64]. These results, along with the impacts of PU and PE on BI, align with the reasoning that older adults are deterred from DCT by a lack of motivation, a lack of interest, or negative content [52]. That is, personal preferences [55] rather than involuntary exclusion determine DCT uptake by older adults. Based on our findings, we suggest a revised TAM specific to older adults, which expands PU and PEU to include perceptions of need, interest, reward for effort, content, situational circumstances, and the impacts of these factors on BI and AU.

This enhanced picture of TAM can be further expanded by privacy and security concerns and situational contexts (in our case, the COVID-19 pandemic) which, although are not included as common constructs of TAM, directly impact BI and AU. The situational context of the pandemic can also be viewed as akin to having restricted life-space mobility, and analogous circumstantial changes that impact mobility would have had a similar impact as the one observed. These additional important themes associated with older adults that are excluded in the traditional TAM were a notable finding and may have prevented full use of the features and advantages of DCT.

Despite extensive research on DCT adoption and the factors influencing the decision processes of users, privacy concerns appear to have received little empirical attention, especially in older adults [35]. The older adults in our study were clearly uncomfortable with Facebook/Meta due to perceived privacy and security intrusion. They also voiced general concerns such as not knowing how to manage security on Facebook/Meta and the possibility of information falling into the wrong hands.

These concerns, expressed as negative responses, were exacerbated by an extraordinary amount of negative press coverage during the study period. The privacy calculus perspective [36] argues that individuals anticipate and assess the privacy-related risks against the benefits of information disclosure when needing to provide private information. The conventional view that privacy-related decisions are guided by predictable risk-reward circumstances [40,41] appeared to be abandoned by older adults. The situational nature of privacy-related decisions on apps has been noted by various scholars [50]. Given the unprecedented circumstances of older adults during the pandemic, we believe that this situational perspective best explains the negative perceptions encountered in this study.

Other privacy and security concerns of the participants were targeting by hackers and online confidence scams. They demonstrated a sense of vulnerability to these threats [42], suggesting that technology use during the pandemic could have been elevated if older adults were empowered to protect their privacy. Protection strategies through which older adults can exert agency over privacy are expected to encourage acceptance.

Overall, we found an increase in the uptake and positive perception of certain DCTs among older adults during the pandemic. Nonetheless, this had its limitations. When examining our qualitative data on the activities, social groups, and organized groups in which participants engaged before the pandemic, we found that DCT was not a suitable alternative to in-person activities. The range and depth of online activities could not fully fulfill the needs of the participants from a social engagement perspective. The participants continued limited engagements with certain organized groups or social activities (eg, online church congregations and a crossword group on Facebook/Meta) but emphasized that these activities offered a less rich experience when compared to in-person activities. There was a strong realization that although videoconferencing improved social connectedness and alleviated loneliness, it was a superficial alternative to in-person interactions. Nevertheless, as already mentioned, email and videoconferencing apps were deemed superior to apps such as Facebook/Meta, suggesting a preference for more immersive DCT tools that might better simulate in-person interactions [23].

Recently, Heitanen et al [93] suggested that eye contact through videoconferencing apps partially offsets the lack of physical presence. Furthermore, videoconferencing offered a more insulated experience from the vagaries of the pandemic than other DCTs. The pandemic-related content in other types of technologies reminded the participants of their restrictions and changes in social interactions and possibly exacerbated anxiety. This discussion reinforces our findings that email and videoconferencing helped alleviate feelings of loneliness and social isolation during the pandemic, with the caveat that videoconferencing can simultaneously highlight the lack of face-to-face interactions but also is probably the best alternative to in-person connection. On the flip side, Facebook/Meta, with its unsolicited content, did little to enhance the social connectedness of users and could also have heightened their anxiety.

The results of this study confirmed 3 aspects related to the use and acceptance of DCT by older adults during the pandemic. First, the pandemic altered the digital profiles of older adults, both by increasing and by changing the usage of DCT. These changes were dominantly driven by communication maintenance, information seeking, and access to services such as shopping and health care. The desire to communicate and access information stemmed from the need to reduce social isolation and loneliness. The respondents reacted positively to different types of DCT fulfilling this desire. We also reported a clear rise in PEU and PU, which might signify a significant emotional link between technological resources and older adults. Our study confirmed that BI to use DCT is an important indicator of AU.

Second, our results also identified a series of barriers that deter older adults from using certain DCT types. Factors leading to a lack of adoption were usability issues, complexity, privacy and security concerns, and (in some cases) the content and information found on these apps. Prior research has similarly identified low user experience, usability problems, anxiety, and distrust related to privacy and security as barriers to technology adoption [33,34].

Finally, we suggest that the pandemic influenced older adults’ intention to use DCT. As a situational context, the pandemic strongly impacted all constructs of TAM. Some DCTs were embraced, while others were largely rejected. If the issues and factors preventing the use of specific DCTs (eg, Facebook/Meta) by older adults are resolved, or if new DCTs are designed with older users in mind, uptake would increase further, providing a valuable resource for combating social isolation and loneliness and accessing civic and health care services.

### Strengths and Limitations

Applying the mixed methods approach, we engaged participants through interviews, gaining richer information than can be gained through surveys; meanwhile, validated surveys were administered to guide the interview process. We covered a wide range of factors influencing the uptake and use of DCT by older adults during the COVID-19 pandemic. However, there were several limitations.

First, this approach means that much of the study relied on self-reported data and was therefore vulnerable to recall and social desirability biases. Second, important variables, such as stress, stigma, socioeconomic status, educational attainment, health disparity, health literacy, and discrimination, were omitted. Another principal limitation was the relatively small sample size and geographical area covered (restricted to the United Kingdom), which makes the generalizability of our results difficult.

Participants only included community-dwelling older adults and did not include older adults in nursing/care homes or assisted living. This is likely to skew results as the health issues of non–community-dwelling older adults is likely to impact their interactions and accessibility to various technologies. Furthermore, non–community-dwelling older adults may have different concerns and needs regarding social connectedness.

Finally, many studies contradict our findings on Facebook/Meta. Older adults comprise an increasing proportion of Facebook/Meta users (21.6% of all users are aged over 65 years versus 15% from 5 years ago) [94]. Others have reported that Facebook/Meta improved the sense of social connectedness during the pandemic [95-98]. In these studies, users remained connected to friends and family through Facebook/Meta and staved off social isolation and loneliness. Notably, none of these studies were based in the United Kingdom, and as their samples were small and disparate, the results should be interpreted with some skepticism.

How older adults use and accept DCT cannot be gleaned entirely from a cross-sectional study. Future research should investigate the extent of the effects of and the circumstances under which these effects occur [99]. This study was designed for periodic or postpandemic follow-up of the participants. These weaknesses may be resolved by improving the quality of the empirical results through a longitudinal analysis in a follow-up study.

### Conclusion

This study, despite its limitations, is 1 of the few mixed method studies in this area and provides needed knowledge about changes in the use of and satisfaction with DCT and how its use has evolved. Our results showed that during the COVID-19 pandemic, DCT use increased because users wished to maintain their social connectedness and access to services. The pandemic and social distancing/lockdown measures significantly reduced the psychosocial well-being of older adults and increased their COVID-19–linked anxiety. The digital profile of the acceptance, uptake, and use of technology by older adults was directly impacted by the pandemic. Certain types of DCT emerged as insulators against the adverse effects of the pandemic, whereas other DCT types exacerbated these effects. The study identified PU, PEU, motivational behavior, design, and privacy concerns as themes requiring special attention for older adults. Policy makers should consider these themes when determining mitigating actions against the detrimental consequences of home confinement. Further research should recognize the non-TAM themes as additional elements in an older adult–specific TAM. In recognizing that DCT adoption is largely driven by the need for social connectedness, future studies should attempt to maximize and enable older adult agency in concepts related to design, privacy, security, and determining user requirements for development.

At the time of writing this paper, Facebook/Meta launched an iPad-type device with a Facebook version called Meta Portal, which is especially designed for older adults. The adoption and ease-of-use perception of Meta Portal by older adults during the pandemic would be an interesting avenue of research. This research is also useful to researchers looking at potential benefits of generative artificial intelligence systems that could be availed for crisis responses and targeting loneliness in older adults.

## Abbreviations

AU: actual use
BI: behavioral intention
CAS: COVID-19 Anxiety Scale
DCT: digital communication technology
PE: perceived enjoyment
PEU: perceived ease of use
PU: perceived usefulness
RQ: research question
SNS: social networking sites
TAM: Technology Acceptance Model

## Data Availability

The data sets generated and analyzed during the study are available from the corresponding author upon reasonable request.
